# Spontaneous regression of cervical intraepithelial neoplasia 3 in women with a biopsy—cone interval of greater than 11 weeks

**DOI:** 10.1186/s12885-022-10179-1

**Published:** 2022-10-18

**Authors:** Maria Teresa Bruno, Nazario Cassaro, Gabriele Mazza, Arianna Guaita, Sara Boemi

**Affiliations:** 1 Department of General Surgery and Medical Surgery Specialties, Gynecological Clinic, University of Catania, Catania, Italy; 2 Multidisciplinary Research Center in Papillomavirus Pathology, University of Catania, Catania, Italy; 3 Gynecological Oncology, Humanitas, Catania, Italy; 4 Department of Statistics, Sapienza University of Roma, Rome, Italy

**Keywords:** CIN3 regression, Biopsy, LEEP, HPV genotype

## Abstract

**Background:**

Although there is broad consensus that only a subset of CIN3 will progress to cancer, there is currently no surefire way to predict which CIN3 will regress. Understanding the natural history of CIN3 is important, and finding markers for progression or regression could improve treatment strategies. According to the guidelines of the American Society for Colposcopy and Cervical Pathology of 2006, positive CIN3 p16 in women should be managed with excisional treatment (LEEP). For ethical reasons we cannot fail to treat women with CIN3 in order to study their regression capacity so we conducted a retrospective study to evaluate the regression rate of CIN3 diagnosed with a biopsy by studying the histological result of the cone removed by LEEP. We also investigated age, HPV genotypes and biopsy-cone interval distance as possible regression factors.

**Methods:**

We selected 171 women with a histological diagnosis of positive CIN3 p16 as an entry criterion. All patients underwent LEEP / biopsy. A histological diagnosis of the cone of CIN3 or higher was considered as persistence or progression, the diagnosis of CIN1 or lower was considered as regression of the lesion.

We used out a logistic model to study the probability of spontaneous regression of CIN3 as a function of the patient’s age, the time elapsed between the biopsy and the cone (in weeks) and the HPV genotype.

**Results:**

We found that the spontaneous regression rate of CIN3 was 15,8%, which was strongly associated with the biopsy-cone interval > 11 weeks. Genotype 16, the most represented, was present both in cases of regression (77.8%) and in persistence (83.3%). Regarding age, the estimated odds ratio of the probability of observing a regression in women over 25 years of age was 0.0045 times that of women under 25 years of age (CI: 0.00020, 0.036). Neither age nor viral genotype are significant as predictors of regression.

**Conclusion:**

To wait at least 11 weeks from the biopsy before subjecting the woman to LEEP could prevent unnecessary LEEP procedures, considering also that from CIN3 to carcinoma it takes years before the neoplastic transformation takes place.

**Supplementary Information:**

The online version contains supplementary material available at 10.1186/s12885-022-10179-1.

## Background

Cervical cancer remains a leading cause of cancer death in women around the world, particularly in low-resource areas. Although HPV vaccines have been proven effective [1, 2] high-grade intraepithelial cervical neoplasia (CIN3) is still a problem, even in high-resource areas [3].

The diagnosis and prevention of cervical carcinoma are based on the assumption of Richart [4] who stated that preneoplastic lesions evolve slowly over time and progress through a spectrum of histological differentiation ranging from CIN1, to CIN2, to CIN3. Progression from HPV infection to virus persistence, to the development of high-grade CINs and eventually invasive cervical cancer seems to take, on average, up to about 15 years, although cases of rapid-onset cancers do occur [5–7].

Persistence of specific viral genotypes is associated with a higher risk of developing CIN2 + .

In particular, persistent HPV 16 infection is known as the most significant independent prognostic factor in the progression of cervical lesions [8]. In one of our studies [9] the presence of the HPV16 genotype was associated with a 5 times greater risk of developing a high-grade lesion ( OR = 4.62 95 CI: 3,13–6,82).

The genotypes that confer more persistence with progression are 16, 31 and 33, these are very frequent in Italy, while HPV 16 and HPV 33 are more frequent in the United States, followed by HPV 31, HPV 18 [10, 11].

The natural history of CIN1 lesions is characterized by a high rate of spontaneous regression (80%) [12–14], follow-up data of meta-analyses show that without treatment 15–40% or more of all CIN3 lesions will naturally regress [15] in immunocompetent women.

Therefore not all women with CIN3 will develop cancer, thus more than 50% of women with CIN3 are currently being overtreated [16] with LEEP (Loop Electrosurgical Excision Procedure). In contrast, more recent studies have not found a significant regression rate among CIN3 lesions [17]. Due to ethical reasons we cannot fail to treat women with CIN3 in order to study their ability to regress; we conducted a retrospective study that evaluated the CIN3 regression rate based on diagnostic biopsies of the excised cone. We also investigated age, HPV genotypes and biopsy-cone interval distance as possible regression factors.

## Methods

We studied the clinical files of 183 patients who, from April 2016 to April 2020, had undergone LEEP for CIN3 at our Colposcopy Clinic of the Gynecology Unit, University hospital, Catania, Italy. We selected the women of whom we were able to reconstruct their entire diagnostic therapeutic process from colposcopy, to biopsy, to LEEP and who met the following inclusion criteria: patients with hr-HPV positive test, histological diagnosis of CIN3 to biopsy, positive for p16 protein, a visible lesion on the portio after biopsy, and had been subjected to LEEP. Patients without pathologies of the immune system and not pregnant.

All the data of the women in our database who met the inclusion criteria were retrospectively analyzed in an observational cohort study. The data were analyzed anonymously. This study is conforms to the provisions of the Helsinki Declaration, as revised in 2013. The study protocol was notified, according to the current legislation on observational studies provided by AIFA, to the Ethics Committee of the Catania University Hospital which did not request additions or changes to the protocol. Furthermore, the Ethics Committee of the Catania University Hospital found the consent of the study participants unnecessary as the study concerned only the retrospective review of the medical database.

Only 171 patients met the inclusion criteria and their clinical data were collected, of which: age, HPV genotype, lesion size, histological result of the targeted biopsy and date, date of LEEP and histological examination of the cone and the biopsy- cone interval were calculated. All these patients had the lesion visible on the portio after the diagnostic biopsy to rule out the possibility that the lesion had been completely removed by the biopsy. Each patient underwent colposcopy, targeted biopsy and HPV testing. The histological examination was supported by the search for the p16 protein, which is performed routinely. Histopathological diagnoses were made using WHO criteria. All the cases included in this study showed a p16 positivity at the diagnostic biopsy and were positive for the p16 protein, an immunohistochemical technique that we use routinely. We selected all the cases of CIN3, histological diagnosis that we chose as an entry criterion, compared to the cytological diagnosis [13] of HSIL.

The patients were examined by conventional Pap smear test and colposcopy, in addition, cytological exocervical samples were taken and placed in Thin Prep solution. Samples were sent to the laboratory for DNA extraction and viral DNA genotyping by genetic amplification followed by hybridization with genotype-specific probes capable of identifying most of the HPV genotypes of the genital region [28 high-risk HPV genotypes (16, 18, 26, 31, 33, 35, 39, 45, 51, 52, 53, 56, 58, 59, 66, 68, 73, 82), low risk (6, 11, 40, 43, 44, 54, 70) and undefined risk (69,71,74)]. The commercial method used was the MAG NucliSenseasy system (bioMerieux SA, Marct l’Etoile, France).

Colposcopy was performed using a Zeiss OPM1F colposcope (Carl Zeiss, Jena, Germany) and applying acetic acid and a Lugol iodine solution. Any colposcopic anomaly was classified according to the nomenclature proposed by the International Federation for Colposcopy and Cervical Pathologies (IFCPC) into 3 degrees of increasing abnormalities according to severity: Abnormal Transformation Zone (ATZ) grade 1 (ATZ1) and grade 2 (ATZ2) or cancer. We evaluated the visibility of the squamous-columnar junction and targeted biopsies were taken from the portio. 72.5% (124 cases) of CIN3 lesions involve the three quadrants of the portio, 17.5% (30 cases) two quadrants of the portio, in 13 (7.6%) cases the lesion extends to the entire portio, only 2.3% (4 cases) affects one quadrant of the portio.

The targeted biopsy is performed according to protocol by the colposcopist on the atypical transformation area. In 56% of cases more biopsy samples were performed (2–3) as ATZ2 often affects the three quadrants of the utero portio. The biopsy specimens had an average diameter of about 5 mm. The hemorrhage was not important. The biopsy piece was sent in formalin to the dedicated pathologist. Then it was fixed in paraffin blocks. Doubtful cases were studied by two pathologists, also resorting to a further p16.

In our study the biopsy/cone were revised by two pathologists.

P16 immunohistochemistry was performed and evaluated using the CINTec histological kit (CINTec © INK4_a._ Roche Diagnostics) according to the manufacturer’s protocol. A detailed description of the p16 technique has been published [18].

For the results of p16 we considered two factors: intensity and distribution. The intensity is considered diffuse block, patchy or focal; the distribution may be limited to a lower third, up to the middle third or up to the upper third.

p16 was positive if the samples showed a continuous nuclear or nuclear and cytoplasmic staining of the cells of the basal and parabasal layer of the epithelium, extended to more than a third of the entire epithelial thickness, and exclusively intense localization.

p16 was negative if the samples showed the absence of staining in the epithelium, cytoplasmic staining of isolated cells or small focal cell clusters, and an extent of less than one third of the epithelial thickness.

As per protocol, women diagnosed with CIN3 were given LEEP within 3–4 weeks of the biopsy and immediately after the end of menstruation so that the woman was not pregnant, this also avoids cases of secondary endometriosis, as well as cases of excessive bleeding during surgery that is carried out in day hospital on an outpatient basis, by experienced personnel. The obtained cone or biopsy was examined by the pathologist. The diagnosis of CIN3 or higher was considered as persistence or progression, the diagnosis of CIN1 or lower was considered as regression of the lesion.

We calculated the time interval in weeks between the diagnostic biopsy and LEEP and between the diagnostic biopsy and the second biopsy. Both intervals will be referred to as the biopsy-cone interval.

In accordance with the journal’s guidelines, we will provide our data for the reproducibility of this study in other centers if such is requested.

### Statistical analysis

The analysis was implemented with the Rstudio software (RStudio Team, 2015. RStudio: Integrated Development Environment for R. Boston, MA. Retrieved from http://www.rstudio.com/).

We used out a logistic model to study the probability of spontaneous regression of CIN3 as a function of the patient’s age, the time elapsed between the biopsy and the cone (in weeks) and the HPV genotype, considering the week and age variables as continuous: OUTPUT (log transform coefficients). To better assess the dependence between regression and age, we then considered three age groups (divided according to the 33rd and 66th percentile: “20–29”, “30–34” and “34 + ”), thus considering the age variable as a category.

Therefore, we carried out the logistic model again with the variable “weeks”, we considered two age classes, under 25 years (including 25) and over 25 years.

Subsequently, the presence of a cut-off of the biopsy-cone interval was identified, after which the probability of regression was greater. The logistic model was calculated several times with the only explanatory variable “weeks” considered as categorical. Each implementation of the model was performed by dividing the sample according to the variable “weeks” increasing the cut-off by one week at a time.

## Results

Some patients did not undergo LEEP immediately 3–4 weeks after the biopsy as per protocol, but postponed the surgery for various reasons: work reasons, family, social commitments, health.

**Fig. 1:**
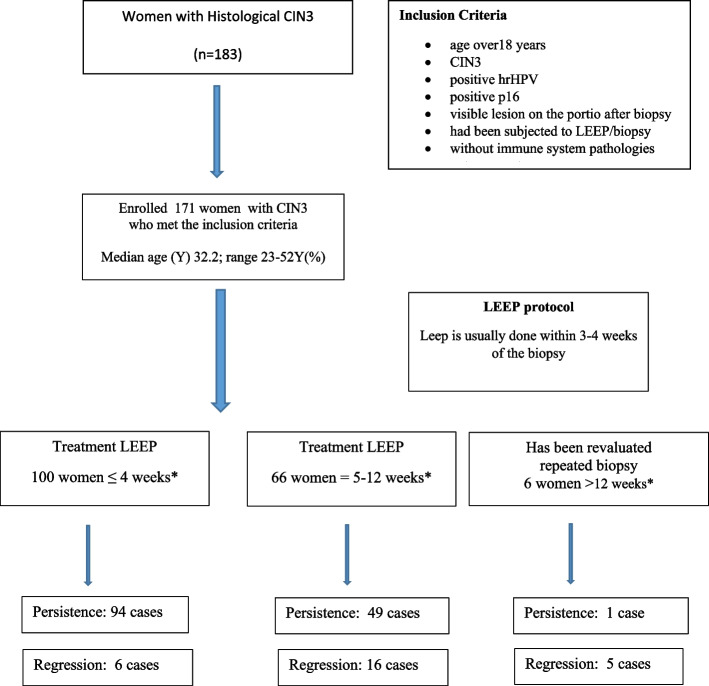
Flow chart of women enrolled in the study

Six women returned for LEEP 16, 18, 19, 21, and 36 weeks after CIN3 diagnosis and are been reevaluated with colposcopy and biopsy. One woman confirmed the diagnosis of CIN3 and underwent LEEP, 5 women showed regression of CIN3 on histological examination and underwent follow-up. The mean age of patients in the study group was 32.2 years (23–52) (Fig. 1).

Spontaneous regression occurred in 15, 8% (CI 95% 10.30- 20,76) (27 cases) of the 171 cases of CIN3 (Table 1). Table 2 shows the estimates of the coefficients of the logistic model applied to the probability of spontaneous regression of CIN3 as a function of the patient’s age, the elapsed time between the biopsy and the cone (in weeks) and the HPV genotype, only the dependence on the variable “weeks” is significant.

**Table 1:** Number of women for week with persistence /regression in the study group

WEEKS	n°	Persistence	Regression
2	8	8	0
3	24	24	0
4	68	62	6
5	6	6	0
6	10	10	0
7	3	2	1
8	14	12	2
9	6	4	2
10	10	6	4
11	10	7	3
12	6	2	4
16	2	1	1
18	1	0	1
19	1	0	1
21	1	0	1
36	1	0	1
	**171**	**144 (84,2%)**	**27 (15,8%)**

**Table 2:** Shows the estimates of the coefficients of the logistic model applied to the probability of spontaneous regression of CIN3 as a function of the patient’s age, the time elapsed between the biopsy and the cone (in weeks) and the HPV genotype, only the dependence on the variable “weeks” is significant

Variable	Estimation of the odds ratio	95% confidence interval	*P*-value
Weeks	1.46	(1.28; 1.71)	3.38*${10}^{-7}$
Age	0.92	(0.81; 1.02)	0.125
HPV_genotype	2.66	(0.79; 8.66)	0.104

As for age, the *p*-value was 0.125 (> 0.05) thus it was not significant, the same for the genotype variable that had a *p*-value of 0.104.

To better assess the dependence between regression and age we considered three age groups (divided according to the 33rd and 66th percentile: “20–29”, “30–34” and “35 + ”) thus considering the age variable as a categorical, however, the coefficients of the model were not significant (Supplementary Table 1).

Considering only two age classes, under 25 years (including 25) and over 25 years, and carrying out the logistic model again with the variable “weeks”, (Table 3) we obtained the output in the Table 4.

**Table 3:** Show the logistic model again with the variable “weeks”, considering only 2 age classes, under 25 years (including 25) and over 25 years

Variable	Estimation of the odds ratio	95% confidence interval	*P*-value
Weeks	1.63	(1.38; 2.04)	6.74*${10}^{-7}$
Age (25 +)	0.0045	(0.00020; 0.036)	1.05*${10}^{-5}$

**Table 4:** Show that the greatest difference between the odds ratio was obtained considering the cut-off at 11 weeks

Variable	Estimation of the odds ratio	95% confidence interval	*P*-value
Weeks 10	10. 05	(3.76; 26.95)	1,38 $*{10}^{-5}$
Weeks (11 +)	22.26	(6.06; 107.21)	1.24*${10}^{-5}$

Therefore, the weeks variable remained significant: increasing the weeks between biopsy and LEEP significantly increased the likelihood of spontaneous regression of CIN3.

Regarding age, the estimated odds ratio of the probability of observing a regression in women over 25 years of age was 0.0045 times that of women under 25 years of age (CI: 0.00020, 0.036). That is, it decreases by 99.6%.

Subsequently, the presence of a cut-off of the biopsy-cone interval was identified, after which the probability of regression was greater (Table 4).

Comparing the estimates of the coefficients of the models carried out, it was observed that the greatest difference between the odds ratio was obtained considering the cut-off at 11 weeks**.**

Supplementary Table 2 shows the results of the viral genotyping of the test sample. Genotype 16, the most represented, was present both in cases of regression (77.8%) and in persistence (83.3%). In the 171 study cases, 141 (82.5%) of patients were positive for genotype16, while 30 (17.5%) patients were positive for other hr-HPVs. Of the patients who had regression, 21 were positive for genotype 16 and only 6 for other hr-HPVs. In our study the viral genotype showed no significant difference. OR:1.43 (CI 0.48–3.74) p 0.488.

## Discussion

Secondary prevention of cervical cancers is based on screening and treatment of cancer precursors. Although there is a broad consensus that only a subset of CIN3 will progress to cancer, there is currently no sure way to predict which CIN3 will not progress to cancer. Studying the progression from CIN3 to cancer is unethical and available data are scarce. New Zealand’s work is the only well-documented series of CIN3 not treated for decades (about 30 years), however, this study examined progression to cancer and not CIN3 regression.

Understanding the natural history of CIN3 is important, and finding markers for progression or regression could improve treatment strategies.

To this end, we evaluated the histological results of LEEP following an initial CIN3 biopsy.

The data under analysis show a positive and significant dependence between the biopsy-cone time interval and the regression rate. We found that the overall rate of spontaneous regression of CIN3 is 15,8%, and that the rate of lesion regression is strongly associated with the biopsy-cone time interval > 11 weeks. There is no significant dependence between regression, age and HPV genotype.

The regression rate in our study corresponds to that of other studies [19–21] and using a logistic model with the three variables together, time (weeks), age and genotype showed us how only the variable time is significant.

For each additional week between biopsy and cone, the odds ratio for the probability of observing a regression increased by 1.46 (= exp (0.37648)), that is, increasing by one week “weeks”, the odds of observing a regression increased by 46% (CI: 1.28, 1.71), with a very significant *p*-value (*p* = 3.38*10^-7).

Regression was observed the longer the biopsy-cone interval, in particular regression was more frequent if the interval between biopsy and LEEP was > 11 weeks.

We had the maximum difference between the odds ratio considering the cut off at 11 weeks: women with the biopsy-cone time greater than 11 weeks, had an estimated odds ratio of the probability of observing a regression 22.26 times greater than women with a time that was less than, or equal to 11 weeks (IC: 6.06. 107.21) with a very significant *p*-value (*p* = 1.24*)${10}^{-5}$.

Even considering the cut-off at 10 weeks, the result was significant, but of less impact: the OR estimated to have a regression was 10.05 times greater than the OR for women with a time ≤ 10 weeks.

Confirming our data is Trimple’s prospective study, which subjected women with CIN2/CIN3 to LEEP 15 weeks after the diagnostic biopsy with a cone regression rate of 28%, even Follen’s study [22] with an interval of 12 months had 50% regression of CIN3 cases, while Munk [23] had 16% regression with an interval of 7.3 weeks.

Important contributions to support this thesis have been made by Trimble who argues that regression is linked to the long cone-biopsy interval and by Munk who provided, with the theory of the immune response induced by the biopsy, the basis for justifying the regression of CIN3 following biopsy. In fact, diagnostic biopsy can induce an inflammatory state and viral antigens come into contact with antigen-presenting agents and induce an effective immune response and subsequent clearance of the lesion.

The effect of diagnostic biopsy on CIN3 regression rates in our study cannot be excluded as is evident from our results, more regression was observed the longer the biopsy-cone interval.

Other factors that we studied related to regression were age and HPV genotype.

As for age, in our logistic model with the three variables together the *p*-value was 0.125 (> 0.05) thus it was not significant (the OR decreases by 9% for each additional year of age, but it was not significant).

We can say that by observing age as a continuous variable, we do not have empirical evidence that demonstrates a correlation between the spontaneous regression of CIN3 and age. However, if we divide the sample into women under 25 and women over 25, the data under review show that women under 25 have a greater spontaneous regression of CIN3 than women over 25 years. All 7 patients observed under 25 years of age had regression within 9 weeks: 6 in the fourth week and one in the ninth week. however, the results should be considered with caution due to the low number of the women (7 units), moreover, for the younger women of the sample we had short times (all verified regression within the first 9 weeks), thus we do not know if after the ninth week the chances of verifying the regression stabilizes. The difference between the numbers of the two groups (7 vs 164) diminishes the power of our conclusions.

There is also no significant dependence between regression and the HPV genotype (coefficient = 2.66(CI:0.79. 8.66), i.e. OR increases by 166%) *p* = 0.4(> 0.05), which means that the probability of observing a regression in patients with hr-HPV genotypes is greater than the probability of observing a regression in patients with genotype 16, but this result is not significant (*p* = 0.4).

Moreover, the difference between the numbers of the two groups (30 for hr-HPV vs 141 for HPV16) decreases the potency of the test.

The strengths of this study include the use of histological diagnosis of CIN3 as an entry criterion, unlike other studies [21] that considered the regression of both CIN2 and CIN3, not considering the different capacity for spontaneous regression. CIN2 “equivocal” lesions increase the overall regression rate and, as a result, regression may have been reported in excess [13]. This led us to consider only histologically confirmed CIN3 and p16 positive cases in the regression investigation.

In addition, other studies used cytological diagnosis of HSIL. Regression rates vary between studies based on cytological and histological diagnoses.

Our study presents some weaknesses including the retrospective nature as ethical reasons do not allow us a prospective study in women with CIN3 lesions. The retrospective nature did not allow for the collection of all data such as the use of oral contraceptive pills, sexual life and the use of drugs that may contribute to regression or persistence. We probably would have had to repeat the HPV test before LEEP especially in cases with a long interval between biopsy and cone to monitor the patient’s viral status. It could be useful to study the viral load of the genotype present in order to identify in the patient who has become HPV negative or with low viral load a relevant factor to predict the absence of CIN3 in the cone. Indeed results of a recent study show that a low viral load before LEEP predicts the absence of lesion in the LEEP sample [24]. Furthermore, the most recent literature data report the ability of some markers (E6/E7 mRNA HPV, methylation index) to be valid as marker of regression of the CIN2 + lesion. Oka et al. are the only authors who have investigated an association between viral DNA methylation and histologically confirmed CIN natural history. In his study (15 cases) there were 8 cases of CIN1/2 with progression to CIN3 and one case of CIN3 that regressed. Methylation rates of the L1 gene were significantly higher in the progression group [25], and it is in this direction that research is shifting, markers of epigenetic effects are among the most studied at the moment [26].

The data under analysis show a positive and significant dependence between the biopsy-cone interval time and the regression rate. We found that the overall rate of spontaneous regression of CIN 3 is 15, 8%, and that the rate of lesion regression is strongly associated with the biopsy-cone time interval > 11 weeks. There is no significant dependence between regression, age and HPV genotype.

## Conclusions

It is clinically important to develop accurate predictors of CIN3 lesion regression. Our results show that among all regression factors analyzed, the biopsy-cone interval greater than 11 weeks is a good regression factor of CIN3. Waiting 11 weeks before subjecting women with CIN3 to LEEP could avoid unnecessary LEEP procedures. These results need to be confirmed by prospective studies.

Although LEEP conization is a safe and widespread technique, several observational studies have highlighted its potential negative effect on fertility, pregnancy outcomes [27, 28] and the sexual health of young women [29].

## Supplementary Information

**Additional file 1: Supplementary Table 1.** To study the dependence between regression and age we divided the truck under study into three age groups (according to the 33rd and 66th percentile: “20-29”, “30-34” and “35+).They are significant.

**Additional file 2: Supplementary Table 2.** Shows the results of the viral genotyping of the test sample.

## Data Availability

The datasets used and/or analyzed during the current study are available from the corresponding author on request.
