# Supplementary material for: Spontaneous regression of cervical intraepithelial neoplasia 3 in women with a biopsy—cone interval of greater than 11 weeks
Source: BMC Cancer. 2022 Oct 18;22:1072. doi: 10.1186/s12885-022-10179-1 (PMC9578209; doi:10.1186/s12885-022-10179-1)
Supplement: Supplementary file 1 — Additional file 1: Supplementary Table 1. To study the dependence between regression and age we divided the truck under study into three age groups (according to the 33rd and 66th percentile: “20-29”, “30-34” and “35+).They are significant. [file 12885_2022_10179_MOESM1_ESM.docx]

| **Variabile** | **Estimation of the odds ratio** | **95% confidence interval** | **p-value** |
| --- | --- | --- | --- |
| Week | 1,45 | 1,27; 1,69 | 4,61$*{10}^{-7}$ |
| Age (30-34) | 0,43 | 0,13; 1,33 | 0.152 |
| Age (35+) | 0,73 | 0,21; 2,35 | 0.601 |

Supplementary Table 1, To study the dependence between regression and age we divided the truck under study into three age groups (according to the 33rd and 66th percentile: "20-29", "30-34" and "35+).They are significant.
