# Supplementary material for: Spontaneous regression of cervical intraepithelial neoplasia 3 in women with a biopsy—cone interval of greater than 11 weeks
Source: BMC Cancer. 2022 Oct 18;22:1072. doi: 10.1186/s12885-022-10179-1 (PMC9578209; doi:10.1186/s12885-022-10179-1)
Supplement: Supplementary file 2 — Additional file 2: Supplementary Table 2. Shows the results of the viral genotyping of the test sample. [file 12885_2022_10179_MOESM2_ESM.docx]

| **genotype** | **n°** | **persistence** | **regression** |
| --- | --- | --- | --- |
| HPV 16 | 141 | 120(83.3%) | 21(77.8%) |
| hr-HPV | 30 | 24(16.7%) | 6(22.2%) |

**S**upplementary Table 2. Shows the results of the viral

genotyping of the test sample.
